# Metabolic Reprogramming, Autophagy, and Reactive Oxygen Species Are Necessary for Primordial Germ Cell Reprogramming into Pluripotency

**DOI:** 10.1155/2017/4745252

**Published:** 2017-07-05

**Authors:** D. Sainz de la Maza, A. Moratilla, V. Aparicio, C. Lorca, Y. Alcaina, D. Martín, M. P. De Miguel

**Affiliations:** Cell Engineering Laboratory, La Paz University Hospital Research Institute IDiPAZ, Madrid, Spain

## Abstract

Cellular reprogramming is accompanied by a metabolic shift from oxidative phosphorylation (OXPHOS) toward glycolysis. Previous results from our laboratory showed that hypoxia alone is able to reprogram primordial germ cells (PGCs) into pluripotency and that this action is mediated by hypoxia-inducible factor 1 (HIF1). As HIF1 exerts a myriad of actions by upregulating several hundred genes, to ascertain whether the metabolic switch toward glycolysis is solely responsible for reprogramming, PGCs were cultured in the presence of a pyruvate kinase M2 isoform (PKM2) activator, or glycolysis was promoted by manipulating PPAR*γ*. Conversely, OXPHOS was stimulated by inhibiting PDK1 activity in normoxic or in hypoxic conditions. Inhibition or promotion of autophagy and reactive oxygen species (ROS) production was performed to ascertain their role in cell reprogramming. Our results show that a metabolic shift toward glycolysis, autophagy, and mitochondrial inactivation and an early rise in ROS levels are necessary for PGC reprogramming. All of these processes are governed by HIF1/HIF2 balance and strict intermediate Oct4 levels. Histone acetylation plays a role in reprogramming and is observed under all reprogramming conditions. The pluripotent cells thus generated were unable to self-renew, probably due to insufficient *Blimp1* downregulation and a lack of Klf4 and cMyc expression.

## 1. Introduction

Primordial germ cells (PGCs) are the embryonic precursors of the gametes. Despite being unipotent stem cells, they share some common features with pluripotent stem cells (PSCs), such as constitutive expression of pluripotency factors *Oct4*, *Sox2*, *Lin28*, and *Nanog* [1–6], and stem cell markers stage-specific embryonic antigen 1 (SSEA1) and tissue-nonspecific alkaline phosphatase (TNAP). Functionally, PGCs are closely related to pluripotency, given that disruptions during their development can give rise to pluripotent, malignant embryonal carcinoma cells (ECCs) [3]. Furthermore, unipotent germ cells differentiate only into gametes but acquire totipotency through fertilization. Thus, germ cells are the only cells that undergo reprogramming under physiological conditions. Probably because of this, PGCs are easily reprogrammed toward pluripotent embryonic germ cells (EGCs) when cultured with basic fibroblast growth factor (bFGF) or trichostatin A (TSA) [7–9]. Previous work from our laboratory has shown that PGCs cultured under hypoxia can give rise to pluripotent cells, termed hypoxia-induced embryonic germ-like cells (hiEGLs), with hypoxia-inducible factor 1 (*HIF1*) *α* as a key factor in the metabolic switch toward glycolysis and subsequent *Oct4* deregulation [10].

In classical induced pluripotent stem cell (iPSC) generation with *Oct4*, *Sox2*, *cMyc*, and *Klf4* [11], reprogrammed cells also undergo metabolic reprogramming, shifting from oxidative metabolism toward a glycolytic phenotype [12, 13]. Moreover, several studies show that metabolic resetting is an active process that takes place during reprogramming [12–14] and that an increase in the expression of glycolytic genes precedes a similar increase in the expression of genes that control self-renewal, suggesting that metabolic resetting has an active and early role in reprogramming. It has also been reported that inhibition of oxidative pathways is important to maintain pluripotency [15], and we demonstrated that hypoxia directly promotes cell reprogramming of in vivo committed cells [10]. This mechanism has also been observed in cancer cells and involves mitochondrial inactivation, which in turn renders low levels of reactive oxygen species (ROS), thus preventing oxidative damage [16, 17]. In fact, glycolysis stimulation or the addition of antioxidants such as ascorbic acid can enhance iPSC derivation [18, 19]. This metabolic shift, mitochondrial inactivation, and ROS clearance have also been observed in our hiEGL cultures when compared with nonreprogrammed PGCs [10]. These data propose a way to induce pluripotency by modifying cell metabolism. Our previous results clearly support this hypothesis and allow further characterization of the importance of cell energy metabolism in the reprogramming process.

## 2. Materials and Methods

*Oct4*-green fluorescent protein (GFP) mice from the C57BL\6 strain were used in this study. PGCs from 8.5 days post coitum (dpc) embryos were isolated and seeded onto a confluent monolayer of mitomycin C-treated Sandoz thioguanine- and ouabain-resistant (STO) cells. STO cells are immortalized mouse embryonic fibroblasts that display stem cell factor on their membrane, allowing for PGC attachment and survival.

The isolation and culture of PGCs were carried out as described in [3], and the medium was changed daily. Briefly, PGCs were cultured over nutritious mitomycin-treated STO cells in DMEM with 15% ES-qualified FBS and supplemented with LIF (Millipore) and SCF (R&D Systems), which are essential for PGC survival and proliferation but do not induce reprogramming, in normoxic or hypoxic (3% O_2_) conditions, and exposed to soluble factors daily. Soluble factors were added in decreasing logarithmic concentrations according to the published literature to establish nontoxic ranges. Factors and final concentrations used were DASA at 0.1 *μ*M, ciglitazone at 0.1 *μ*M, dichloroacetate at 50 *μ*M and 500 *μ*M, 2-methoxyestradiol at 0.1 *μ*M, resveratrol at 0.5 *μ*M, chloroquine at 5 *μ*M, spermidine at 1 *μ*M, ascorbic acid at 50 *μ*g/mL, and valproic acid at 5 mg/mL.

TNAP staining was performed as described in [3]. Colonies of 8 or more cells were regarded as reprogrammed PGCs. A *t*-test was then performed between experimental conditions with 3 technical replicates and at least 3 biological replicates.

Immunofluorescence was performed after fixation using 4% paraformaldehyde at pH 7.4. PGCs were identified by labeling for SSEA-1 (R&D Systems). Double staining together with molecules of interest was performed: phospho-pyruvate dehydrogenase (p-PDH) (Abcam), HIF1*α* (Abcam), HIF2*α* (Abcam), Nanog (Abcam), Klf4 (R&D Systems), cMyc (Santa Cruz Biotechnology), p62 (Abcam), p300 (R&D Systems), H2BacK20 (Abcam), H3acK9 (Abcam), and H4acK5K8,K12,K16 (Abcam). As controls, the mouse embryonic stem cell line E14Tg2a, monkey Cos7, human Hela, and mouse NIH3T3 cell lines were used. Images were obtained by confocal microscopy.

To show mitochondrial activity, flow cytometry was performed after addition of a mitochondrial JC-1 fluorescent probe or Cell ROX Green probe (Life Technologies) and subsequent fixation as previously described [10] or with live cells to simultaneously detect Oct4-GFP levels.

Cell sorting was also performed based on this design to isolate PGCs and to extract RNA for specific gene expression analysis, using the RNeasy Mini Kit (Qiagen). RNA was converted to cDNA using the High Capacity cDNA Reverse Transcription Kit (Applied Biosystems), which was then preamplified using TaqMan PreAmp Master Mix (Applied Biosystems) to reach enough of a sample before performing qPCR, using *β-actin* as the reference gene. Primers *PPAR* Forward 5′-3′: tggggatgtctcacaatgc, Reverse 3′-5′: tgggttcagctggtcgata, *Blimp* Forward 5′-3′: gtctgtgccaagacgttcg, Reverse 3′-5′: gaaaggccgttctccactg, *Bnip3* Forward 5′-3′: aaaacagcactctgtctgagga, Reverse 3′-5′: gcttcgggtgtttaaaaagga, *β-actin* Forward 5′-3′: ctgtattcccctccatcgtg, Reverse 3′-5′: aggagtccttctgacccattc. qPCR conditions were 10 min at 95°C and then 10 cycles of 15 s at 95°C and 4 min at 60°C in a Biometra TPersonal.

To demonstrate pluripotency, embryoid bodies (EBs) and three germ layer differentiation were obtained and demonstrated from reprogrammed PGCs as in [10]. The antibodies chosen were antialbumin (Dako) for endoderm, antivimentin (Dako) for mesoderm, and anticytokeratins AE1/AE3 (Dako) for ectoderm demonstration by immunofluorescence.

Electron microscopy was used for observing autophagic processes. PGCs and STO cells were cultured in Permanox-covered culture wells (Cultex) and embedded in epoxy resin. Standard ultrathin sections stained with uranyl and Pb were observed on an electron microscope. In addition, autophagic vacuoles were immunolabeled by anti-p62 antibody (Abcam), and confocal imaging and image analysis (ImageJ) were used to count the number of autophagic vacuoles in confocal stacks. At least 30 cells were counted per condition. Data are shown as number of autophagic vacuoles per whole cell.

Statistical significance in cultures was assessed by Student's *t*-test with Fisher modification in at least 3 separate experiments, each performed in triplicate. EBs and spontaneous differentiation were performed at least 3 times. Flow cytometry results were assessed by parametric F-Snedecor test analysis. All significances were considered at *p* ≤ .05.

## 3. Results

### 3.1. Glycolysis Is Necessary but Not Sufficient for PGC Reprogramming

**Figure 1:**
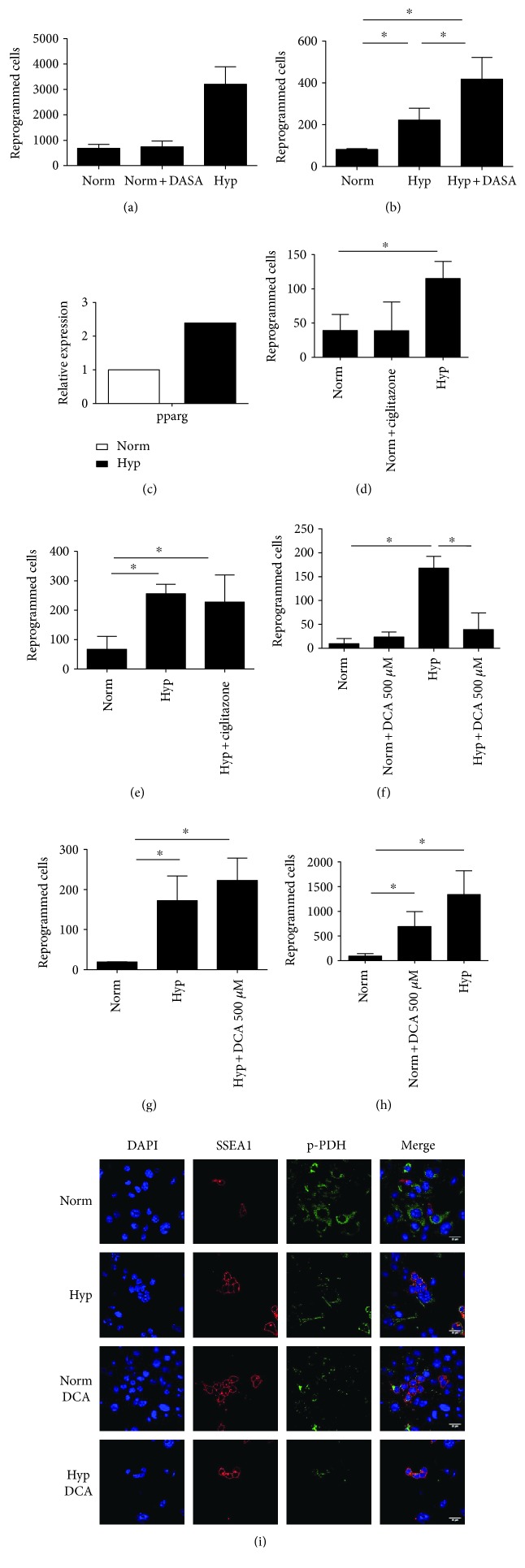
(a) Number of reprogrammed cells in PGC cultures supplemented with DASA (0.1 *μ*M) for 7 days in normoxia. No differences are observed with respect to normoxia. (b) Number of reprogrammed cells in PGC cultures supplemented with DASA (0.1 *μ*M) for 7 days in hypoxia. DASA causes a synergistic effect with hypoxia. (c) Relative expression of PPAR*γ* in PGCs cultured under normoxia or hypoxia. Results are shown normalized with respect to normoxia. (d and e) Number of reprogrammed cells in PGC cultures supplemented with PPAR*γ* agonist ciglitazone (0.1 *μ*M) for 7 days in (d) normoxia or (e) hypoxia. No differences are observed by ciglitazone addition with respect to controls. (f) Number of reprogrammed cells in PGC cultures supplemented with DCA at a high dose (500 *μ*M) for 7 days in normoxia and in hypoxia. DCA prevents hypoxia-induced reprogramming and it has no effect in normoxia. (g and h) Number of reprogrammed cells in PGC cultures supplemented with DCA at a low dose (50 *μ*M) for 7 days. (h) DCA in normoxia is capable of reprogramming, (g) whereas it has no further effect in hypoxia. Asterisks show significance at *p* < .05. (i) Immunofluorescence against phosphorylated PDH (p-PDH) in normoxia (norm) and hypoxia (hyp) with and without 50 *μ*M DCA for 4 days. From left to right: in blue, nuclei stained with DAPI; in red, PGCs detected as SSEA1^+^ cells; in green, p-PDH. Scale bars represent 25 microns.

In a previous study, we demonstrated that hypoxia reprograms PGCs into pluripotency and that this action is mediated by *HIF1* [10]. Because HIF1 exerts a myriad of actions by upregulating several hundred genes, to ascertain if the metabolic switch toward glycolysis is solely responsible for reprogramming, we cultured PGCs in the presence of DASA, a pyruvate kinase M2 isoform (PKM2) activator. We thus hypothesized that an increase in the glycolytic flux would promote reprogramming. DASA was not able to mimic hypoxia in inducing PGC reprogramming (Figure 1(a)), suggesting that additional effects of HIF1 are required. However, DASA exerted a synergistic effect together with hypoxic culturing (Figure 1(b)), suggesting that even in a glycolytic-prone metabolism promoted by hypoxia, an increase in the glycolytic flux further improves reprogramming efficiency.

Similarly, we then promoted glycolysis by manipulating *PPARγ*, a master regulator of metabolism. PPAR*γ* can increase glucose uptake inducing GLUT4 expression, contributes to glycolysis initiation by inducing glucokinase expression, and inhibits the tricarboxylic acid cycle by enhancing pyruvate dehydrogenase kinase (*PDK*) 4 expression. Accordingly, we found an increase in PPAR*γ* expression under hypoxia (Figure 1(c)), which indicates that a metabolic reprogramming is taking place during PGC reprogramming. The use of ciglitazone, a PPAR*γ* agonist, in normoxia did not lead to PGC reprogramming (Figure 1(d)), indicating that PPAR*γ* alone is unable to provoke a metabolic shift that, in turn, induces PGC reprogramming. Also, ciglitazone did not synergize with hypoxia (Figure 1(e)), consistent with the observed upregulation of *PPARγ* by hypoxia alone.

Conversely, we stimulated oxidative phosphorylation (OXPHOS) by inhibiting PDK1 activity in hypoxia by dichloroacetate, a PDK inhibitor. We hypothesized that inhibition of PDK would inhibit PGC reprogramming. As expected, dichloroacetate (DCA) impaired hypoxia-induced PGC reprogramming (Figure 1(f)), suggesting that a glycolytic metabolism is required for reprogramming, whereas it had no effect in normoxia (Figure 1(f)). Nonetheless, when a 10-time lower dose of DCA was used, no impairment of reprogramming in hypoxia was observed (Figure 1(g)), and most importantly, a significant increase in the number of reprogrammed cells was shown in normoxia (Figure 1(h)).

A possible explanation is that in low-DCA dose conditions, a glycolytic signature is observed in PGCs, as confirmed by the fact that in such cultures PDH is phosphorylated and thus inactive; thus, the low dose of 50 *μ*M DCA does not inhibit PDK1, and p-PDH can still be found in cultures under normoxia or hypoxia in combination with DCA (Figure 1(i)).

**Figure 2:**
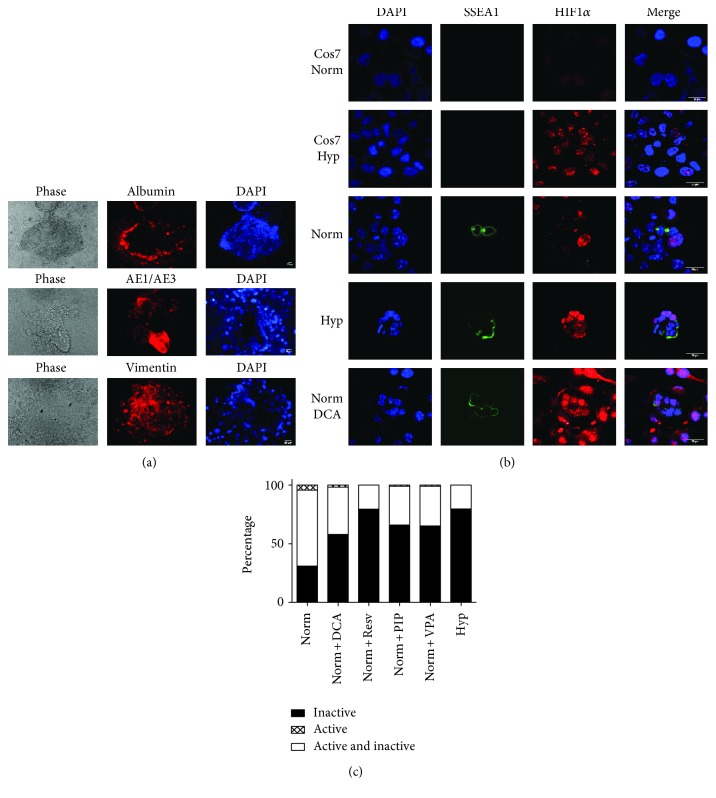
(a) EB formation and spontaneous differentiation of reprogrammed PGCs with a low dose of DCA (50 *μ*M) in normoxia. The markers of endoderm, ectoderm, and mesoderm are, respectively, albumin, AE1/AE3 cytokeratins, and vimentin. Scale bars correspond to 25 *μ*m. (b) Confocal microscopy images for immunofluorescence against SSEA1 and HIF1*α* in PGC cultures. Samples include Cos7 cells cultured in normoxic conditions as a negative control and in hypoxia as a positive control. Images also show PGCs cultured in normoxia (norm), hypoxia (Hyp), and dichloroacetate (50 *μ*M) in normoxia (norm + DCA) for 48 h. Scale bars correspond to 25 *μ*m. (c) Flow cytometry data of PGC cultures showing the percentage of SSEA1^+^ cells displaying green signal from the JC-1 probe (inactive mitochondria), red signal (active mitochondria), or bivalent (both types). An increase in inactive mitochondria in detriment of bivalent mitochondria is observed in normoxia together with either low DCA dose (50 *μ*M), resveratrol (0.5 *μ*M), or VPA (5 mg/mL) and in hypoxia after 5 days with respect to normoxic cultures.

We next investigated how a low dose of DCA induces reprogramming. First, we confirmed the pluripotent capacity of these cells by embryoid body generation and three germ layer spontaneous differentiations. Positive staining for the three germ layers proved that, as with hiEGLs, DCA-reprogrammed cells display pluripotency at least in vitro (Figure 2(a)).

We next explored the possible stabilization of HIF1 in DCA cultures. In fact, HIF1 was stabilized in PGCs by a low dose of DCA, similarly to hypoxic cultures (Figure 2(b)), revealing a possible and similar mechanism of PGC reprogramming by hypoxia and DCA.

We hypothesized that HIF1 inhibition would impair PGC reprogramming under hypoxia. For that purpose, we used a HIF1 inhibitor, 2-methoxyestradiol (2-ME), expecting to see an impairment in reprogramming. The 2-ME showed no effect at low doses and shown to be toxic to PGCs at high doses (not shown), suggesting that HIF1 is essential not only for reprogramming but also for PGC survival in hypoxia.

We then explored the possible effects of a low dose of DCA in mitochondrial activity. In normoxia, most PGCs show both inactive and active mitochondria, followed by a large percentage of PGCs with inactive mitochondria and a minimal amount with active ones. Cells with DCA showed an increase in the percentage of inactive mitochondria with respect to normoxia, as demonstrated by JC-1 labeling and flow cytometry (Figure 2(c)). This decrease in mitochondrial activity is related to the glycolytic signature of PGC metabolism under low DCA and points to the possible involvement of autophagic mechanisms in PGC reprogramming.

### 3.2. Autophagy Is Necessary but Not Sufficient for PGC Reprogramming

**Figure 3:**
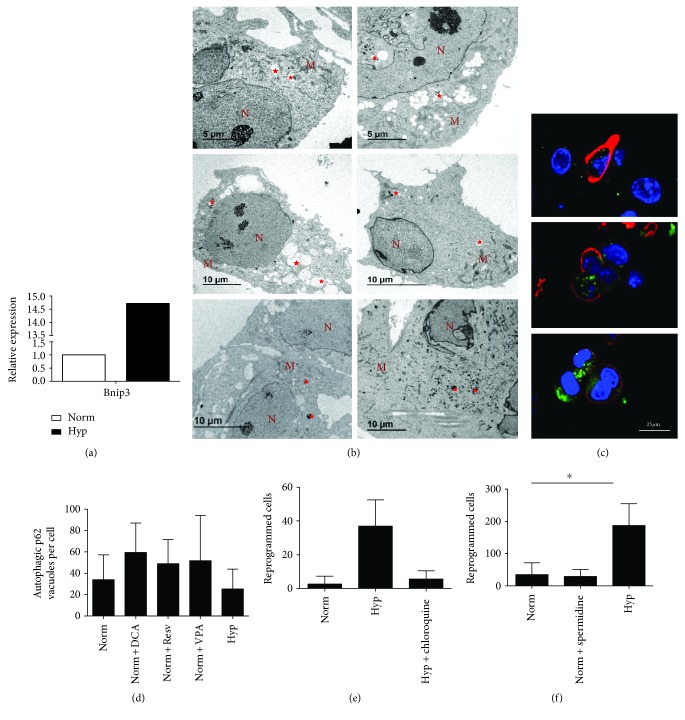
(a) Relative expression of *Bnip3* gene carried out by quantitative PCR from PGCs cultured under normoxia or hypoxia. Results are shown normalized with respect to normoxia. (b) Electron microscopy photographs of PGCs in normoxia (top row), hypoxia (second row), and normoxia with 50 *μ*M DCA (bottom). Left column day 3 and right column day 6 cultures. Autophagic vacuoles are labeled with red stars. M: mitochondria. N: nucleus. (c) Confocal microscopy merge images for immunofluorescence against SSEA1 in red (PGCs) and p62 autophagic vacuoles in green in cytoplasms of PGC cultures subjected to normoxia (top row), hypoxia (second row), and normoxia with 50 *μ*M DCA (bottom) for 3 d. Nuclei are seen in blue with DAPI. Scale bars correspond to 25 *μ*m. (d) Quantification of p62-positive autophagic vacuoles in confocal stacks shows a nonstatistically significant increase with respect to normoxia. (e) Inhibition of autophagy with chloroquine (5 *μ*M) for 7 days prevents hypoxia-induced reprogramming. (f) Induction of autophagy with spermidine (1 *μ*M) in normoxia for 7 days does not induce reprogramming. Asterisks show significance at *p* < .05.

We next explored the possibility that autophagy, and specifically mitophagy, has a direct role in PGC reprogramming exerted by both hypoxia or normoxia and a low dose of DCA. In fact, *Bnip3*, a key regulator of mitophagy and a HIF1 target, demonstrated a 14-fold increased expression in hypoxia in comparison to normoxia by qRT-PCR (Figure 3(a)). As expected, we confirmed the presence of mitophagic vacuoles in normoxia and also in hypoxia and DCA conditions by electron microscopy (Figure 3(b)). DCA (and the rest of reprogramming conditions, see below) caused a slight increase in the number of p62-positive autophagic vacuoles at day 3 (Figures 3(c) and 3(d)), although it was not significant due to high SD between cells.

To demonstrate the role of autophagy in PGC reprogramming, we next prevented autophagy in PGC cultures subjected to hypoxia with the autophagy inhibitor chloroquine. In fact, hypoxia-induced reprogramming was prevented (Figure 3(e)), demonstrating that autophagy is required for reprogramming. When we induced autophagy with spermidine, no reprogramming was achieved (Figure 3(f)), suggesting that autophagy alone is not sufficient for PGC reprogramming.

**Figure 4:**
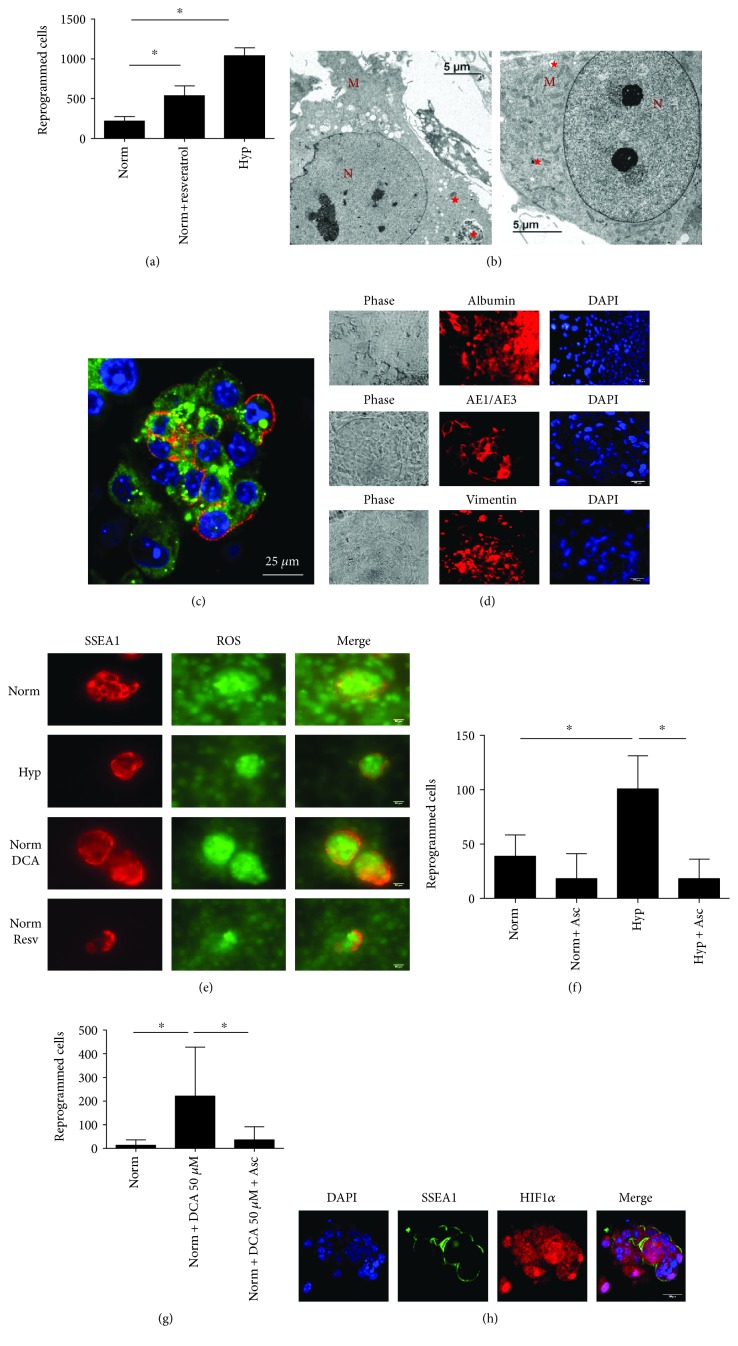
(a) Number of reprogrammed PGCs in conditions of normoxia, normoxia and resveratrol (0.5 *μ*M), and hypoxia for 7 days. Resveratrol induces PGC reprogramming. Asterisks show significance at *p* < .05. (b) Electron microscopy photographs showing 0.5 *μ*M resveratrol-induced mitophagy at day 3 (left) and day 6 (right). Autophagic vacuoles are labeled with red stars. M: mitochondria. N: nucleus. (c) Confocal microscopy merge image for immunofluorescence against SSEA1 in red (PGCs) and p62 autophagic vacuoles in green in PGC cytoplasms of cultures subjected to normoxia with 0.5 *μ*M resveratrol for 3 d. Nuclei are seen in blue with DAPI. Scale bars correspond to 25 *μ*m. (d) Embryoid body formation and spontaneous differentiation of reprogrammed cells into cells of the three germ layers by resveratrol (0.5 *μ*M). (e) Immunofluorescence images of ROS levels in PGC (SSEA1+) cultures exposed to normoxia, hypoxia, normoxia and low DCA dose (50 *μ*M), and normoxia and resveratrol (0.5 *μ*M) for 20 h. Scale bars correspond to 20 *μ*m. (f) Number of reprogrammed PGCs in conditions of normoxia, normoxia and ascorbic acid (50 *μ*g/mL), hypoxia, and hypoxia and ascorbic acid (50 *μ*g/mL), for 7 days. Ascorbic acid does not induce reprogramming in normoxia and prevents hypoxia-induced reprogramming. (g) Number of reprogrammed PGCs in conditions of normoxia, normoxia and DCA (50 *μ*M), normoxia and DCA (50 *μ*M), and ascorbic acid (50 *μ*g/mL) for 7 days. Ascorbic acid prevents reprogramming induced by DCA. Asterisks show significance at *p* < .05. (h) Confocal microscopy images for immunofluorescence against SSEA1 and HIF1*α* in PGC cultures subjected to resveratrol for 48 h. Controls are the same as in Figure 2(b). Scale bars correspond to 25 *μ*m.

However, when we induced mitophagy with resveratrol, reprogramming was achieved in normoxia (Figure 4(a)). Mitophagy was also observed by electron microscopy (Figure 4(b)), and the number of p62-positive autophagic vacuoles was slightly increased respect to normoxia at day 3 (Figures 3(d) and 4(c)). There was also an increase in the percentage of cells with inactive mitochondria, higher than that observed with DCA, and similar to that achieved under hypoxic conditions (Figure 2(c)). Pluripotency induction by resveratrol was also confirmed by EB formation and spontaneous differentiation into cells of the three germ layers (Figure 4(d)).

### 3.3. A Precise Level of ROS Is Required for PGC Reprogramming

In addition to inducing autophagy similarly to spermidine, resveratrol is also a potent antioxidant capable of reducing ROS levels. In fact, after 20 h of culture, we detected lower ROS levels in PGCs exposed to resveratrol with respect to normoxia, and also, we detected ROS in DCA-exposed cultures (Figure 4(d)). To explore the possibility of inducing pluripotency in PGCs by reducing ROS levels, we cultured PGCs in normoxia in the presence of the potent antioxidant ascorbic acid (vitamin C). The ascorbic acid did not have a reprogramming effect (Figure 4(e)), suggesting that the sole reduction of ROS is not capable of inducing reprogramming. However, when we tested whether ROS clearance could disrupt reprogramming, we observed that ascorbic acid is capable of preventing hypoxia-induced reprogramming (Figure 4(e)), suggesting that a specific level of ROS is necessary for PGC reprogramming. Also, in DCA-induced PGC reprogramming, ROS removal impaired the event of cell reprogramming as well (Figure 4(f)), further supporting our hypothesis that a rise in ROS is necessary for reprogramming.

In addition to its mitophagy induction and antioxidant and ROS clearance actions, resveratrol also induces sirtuins, which in turn might stabilize HIF1, and thus the rest of HIF1 actions. In fact, HIF1 immunofluorescence in resveratrol-cultured PGCs demonstrated HIF1 stabilization (Figure 4(g)). Thus, resveratrol induces pluripotency in PGCs by simultaneously acting on mitophagy, ROS levels, and glycolysis induction via HIF1a stabilization.

### 3.4. PGC Reprogramming Pathways Converge in HIF1 Stabilization and Oct4 Deregulation

**Figure 5:**
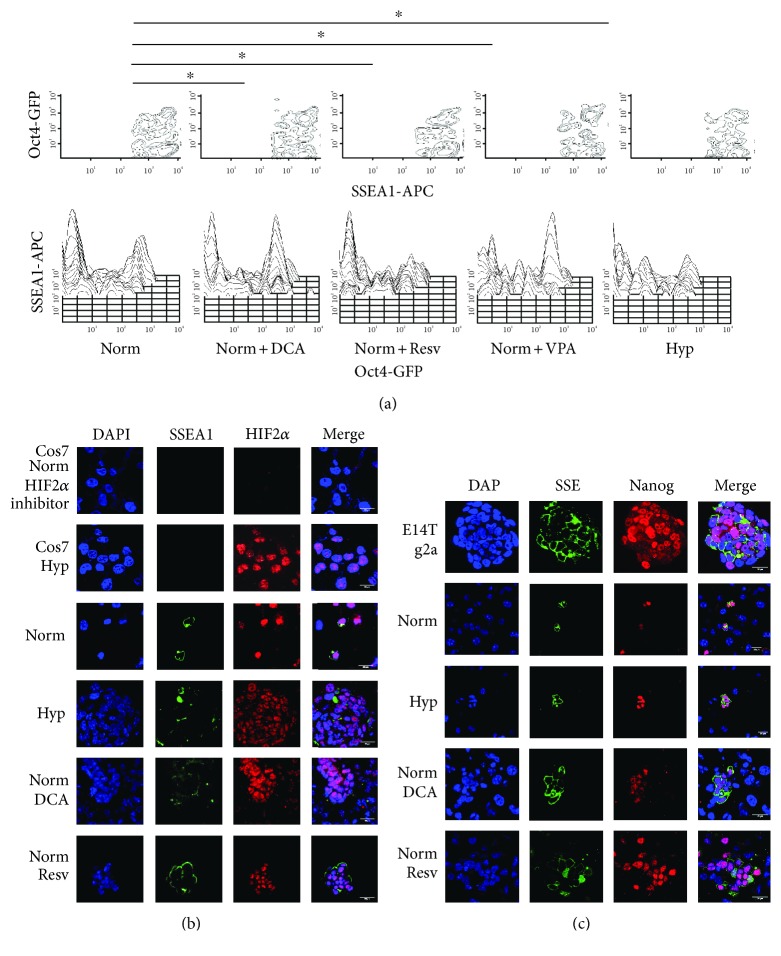
(a) Oct4-GFP flow cytometry in PGC cultures under normoxia, normoxia and 50 *μ*M DCA, normoxia and 0.5 *μ*M resveratrol, normoxia and 5 mg/mL VPA, and hypoxia for 4 days. Normoxic cultures show two separate populations with high and low Oct4-GFP levels, whereas reprogramming cultures under normoxia with DCA, resveratrol and VPA, and hypoxia show a statistically significant appearance of a population with intermediate Oct4 levels. Asterisks show significance at *p* < .05. (b) HIF2 immunofluorescence in normoxic, hypoxic, normoxia + DCA (50 *μ*M), and normoxia + resveratrol (0.5 *μ*M) cultures showing positive reaction in SSEA1+ cell (PGC) nuclei under every condition. (c) Nanog immunofluorescence in normoxic, hypoxic, normoxia + DCA (50 *μ*M), and normoxia + resveratrol (0.5 *μ*M) cultures showing positive reaction in SSEA1+ cell (PGC) nuclei under every condition.

In PGC hypoxic cultures, HIF1 stabilization promotes deregulation of Oct4 levels, which in PGCs are already high, lowering them to promote reprogramming [10]. To ascertain whether reprogramming in normoxia by low DCA and resveratrol uses the same mechanism, we performed flow cytometry for Oct4-GFP in such cultures (Figure 5(a)). In fact, both DCA and resveratrol showed a similar pattern of Oct4 expression to hypoxia and statistically different from normoxia, suggesting that the same Oct4 deregulating mechanism is being used.

Because HIF2 has been described to be the HIF responsible for Oct4 regulation, we studied the expression of HIF2 in our PGC cultures in order to ascertain whether the HIF1/HIF2 equilibrium could be responsible for PGC reprogramming. HIF2 expression was found in all the conditions studied, even in normoxia (Figure 5(b)), suggesting that HIF1 is responsible for PGC reprogramming. Interestingly, both HIF1 and HIF2 expression disappeared after 6 days in every condition studied (not shown).

We also studied the possible effects of the reprogramming factors in *Nanog* expression, given that *Nanog* has been demonstrated to be a master regulator of pluripotency that is also expressed in PGCs. Nanog was expressed in every condition studied, as expected (Figure 5(c)), adding to the evidence that reprogramming toward pluripotency has been achieved. These results also agree with the reduction of Blimp1 expression, a gene related to germ cell identity that downregulates in hypoxia-induced reprogramming (not shown).

**Figure 6:**
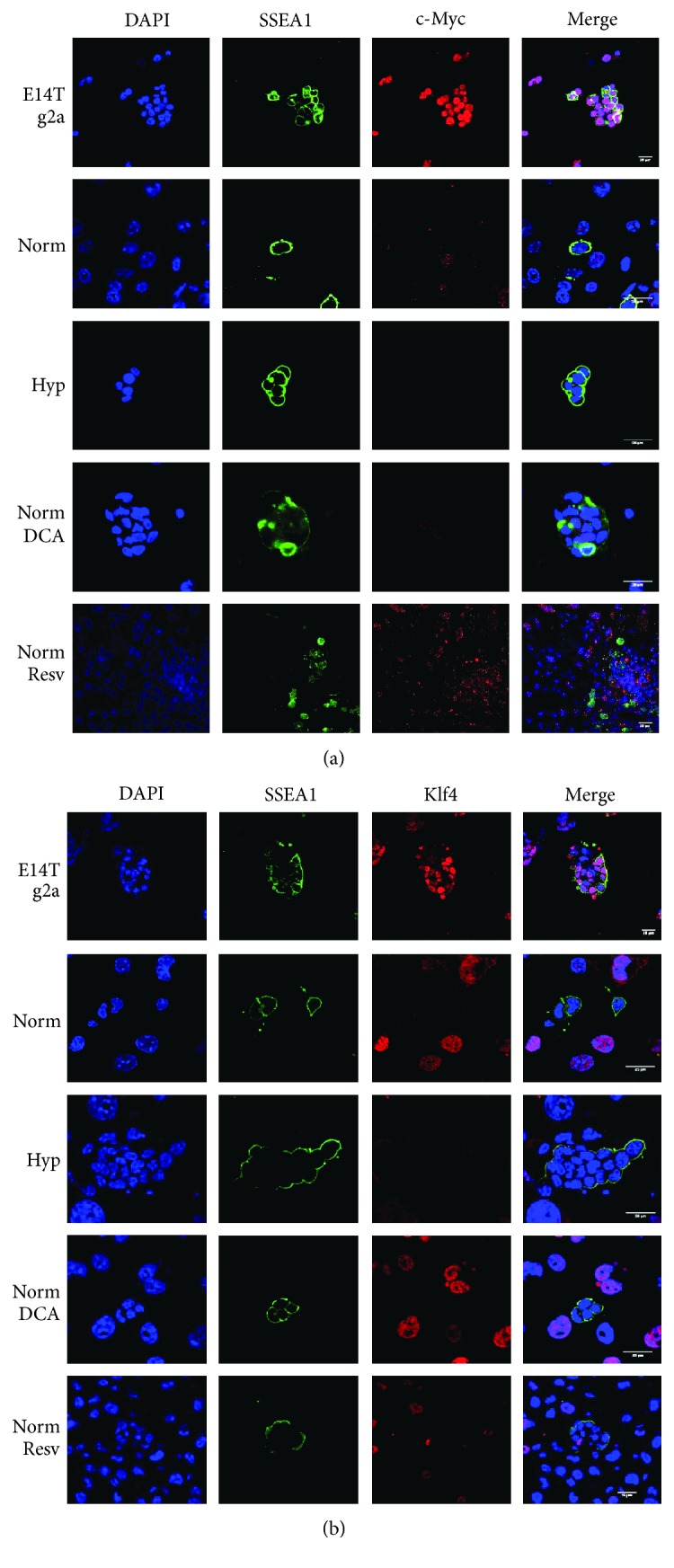
(a) cMyc immunofluorescence in normoxic, hypoxic, normoxia + DCA (50 *μ*M), and normoxia + resveratrol (0.5 *μ*M) cultures showing lack of reaction in SSEA1+ cell (PGC) nuclei under every condition. (b) Klf4 immunofluorescence in normoxic, hypoxic, normoxia + DCA (50 *μ*M), and normoxia + resveratrol (0.5 *μ*M) cultures showing negative reaction in SSEA1+ cell (PGC) nuclei under every condition. Positive controls are the ES cell line E14Tg2. Scale bars correspond to 25 *μ*m.

Similarly to hypoxia, DCA and resveratrol did not induce self-renewal in induced PGCs, leading to the generation of a stable cell line. For that reason, we investigated the expression of Klf4 and cMyc in cultures under DCA and resveratrol. Neither factor was able to upregulate either gene (Figures 6(a) and 6(b)), providing an explanation for the lack of self-renewal of the reprogrammed cells similar to hypoxia.

### 3.5. Epigenetic Control of PGC Reprogramming

**Figure 7:**
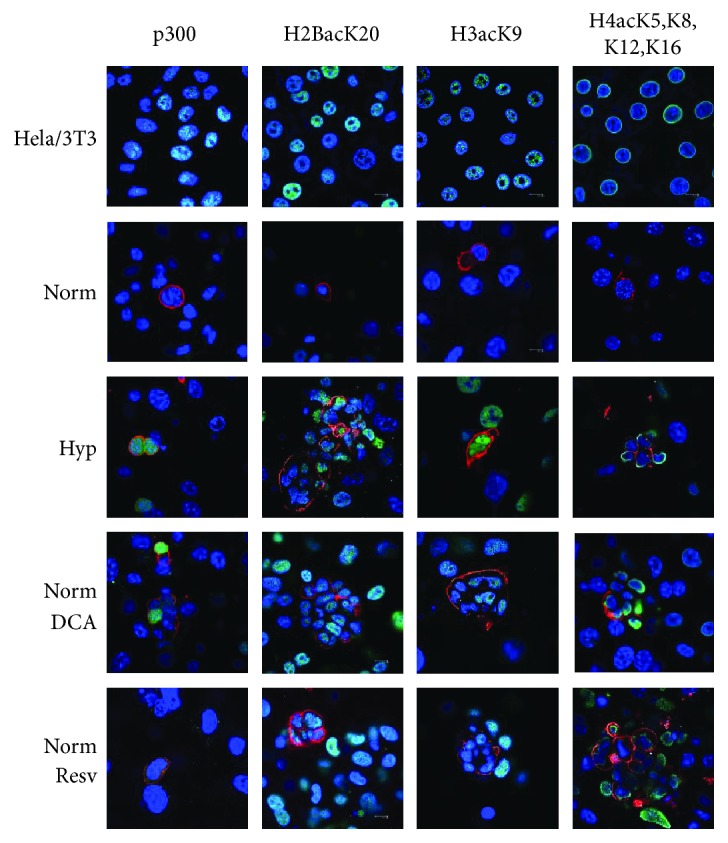
Confocal merge images of p300, H2BacK20, H3acK9, and H4acK5K8,K12,K16 immunofluorescence (in green in the nuclei) in normoxic, hypoxic, normoxia + DCA, and normoxia + resveratrol cultures, showing positive reaction in SSEA1+ cells (PGCs, membranes in red). Blue shows nuclei with DAPI stain. Colocalization is seen as light blue. p300 and histone acetylations were negative in the nuclei of normoxic SSEA1+ cells (PGCs) and positive under reprogramming conditions (DCA at 50 *μ*M and resveratrol at 0.5 *μ*M, all conditions maintained for 48 h). Positive controls are the human untreated Hela cell line for p300 and the NIH3T3 mouse cell line subjected to the histone deacetylase inhibitor Na^+^ butyrate at 0.25 *μ*M for 16 hours for histone acetylations. Scale bars correspond to 100 *μ*m in every photograph.

To characterize the implication of epigenetics in the reprogramming with DCA and resveratrol in normoxic conditions, *p300* (*CREB-binding protein-CBP-coactivator*) acetyltransferase expression was investigated. p300 was expressed in the PGCs' nuclei in all reprogramming conditions (Figure 7). Actual histone acetylation was evaluated by immunocytochemistry against H2BacK20, H3acK9, and H4acK5K8,K12,K16. Our results show that whereas histones H2B, H3, and H4 are nonacetylated in normoxic PGCs, they become acetylated both in normoxic DCA and resveratrol-supplemented cultures as early as 48 h of treatment (Figure 7).

**Figure 8:**
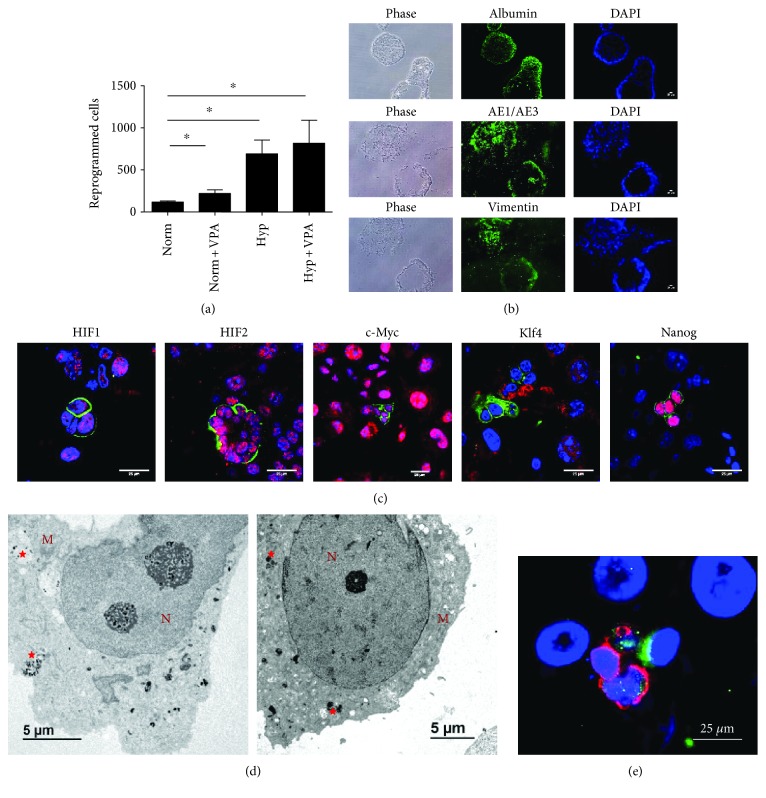
(a) Number of reprogrammed PGCs in conditions of normoxia, normoxia and valproic acid (VPA), hypoxia, and hypoxia and VPA (5 mg/mL) for 7 days. VPA induces PGC reprogramming in normoxia but does not synergize with hypoxia. Asterisks show significance at *p* < .05. (b) EB formation and spontaneous differentiation of reprogrammed cells into cells of the three germ layers by VPA in normoxia (5 mg/mL). Markers of endoderm, ectoderm, and mesoderm are, respectively, albumin, AE1/AE3 cytokeratins, and vimentin. Scale bars correspond to 25 *μ*m. (c) Confocal microscopy merge images for immunofluorescence against SSEA1 in green (PGCs), and HIF1*α*, HIF2*α*, cMyc, Klf4, and Nanog in red in nuclei of PGC cultures subjected to 5 mg/mL VPA for 48 h. Colocalization is seen as bright pink. Controls are the same as in Figure 2(b). Scale bars correspond to 25 *μ*m. (d) Electron microscopy photographs showing VPA-induced mitophagy at day 3 (left) and day 6 (right). Autophagic vacuoles are labeled with red stars. M: mitochondria. N: nucleus. (e) Confocal microscopy merge image for immunofluorescence against SSEA1 in red (PGCs) and p62 autophagic vacuoles in green in cytoplasms of PGC cultures subjected to normoxia with 5 mg/mL VPA for 3 d. Nuclei are seen in blue with DAPI. Scale bars correspond to 25 *μ*m.

As histone acetylation was positive in all the reprogramming conditions in normoxia, and also in hypoxia alone, we explored the possibility that manipulation of histone acetylation was in fact capable of reprogramming directly. We made use of valproic acid (VPA), a wide histone deacetylases inhibitor. We demonstrate that VPA is capable of PGC reprogramming in normoxia (Figure 8(a)), whereas no further effect was seen in hypoxic conditions (Figure 8(a)). Reprogramming toward pluripotency was characterized by EB formation (Figure 8(b)). Similarly to DCA and resveratrol, Oct4 expression levels were also altered by VPA (Figure 5(a)). Mitochondrial activity was diminished as well with respect to normoxia (Figure 2(c)). HIF1 and HIF2 expression were confirmed as well (Figure 8(c)). VPA-treated cultures were positive for Nanog and negative for Klf4 expression as assessed by immunofluorescence (Figure 8(c)). Interestingly, VPA-treated cultures were positive for cMyc (Figure 8(c)). Autophagy was also slightly increased at day 3 as shown by electron microscopy (Figure 8(d)) and p62 immunolabeling (Figure 8(e)) and quantification of autophagic vacuoles (Figure 3(d)).

## 4. Discussion

Our results show that a mere soluble factor is able to induce acquisition of pluripotency in PGCs, as we had previously shown using only hypoxia [10]. Previous work from our laboratory showed that PGCs and EGCs display a differential expression profile, which differs in energetic metabolism genes [10]. Our results indicated an essential role of *HIF1* in PGC reprogramming, due to direct metabolic reprogramming that drives pluripotency acquisition. It is known that HIF1 induces the glucose transporter (GLUT), *pyruvate dehydrogenase kinase 1*, *lactate dehydrogenase A*, and *hexokinase* expression [20–26]. HIF stabilization through prolyl hydroxylase inhibition was sufficient to induce PGC reprogramming [10]. Our results show that PKM2 activation using soluble factor DASA and the consequent boost in glycolytic flux promote a higher number of reprogrammed cells in hypoxia, but not in normoxia. *PKM2* expression can be enhanced by HIF1 or PPAR*γ* [27, 28], thus it is probable that a PKM2 activation in hypoxia (in which it is likely that PKM2 is operating and where there certainly is a glycolytic profile) contributes to more reprogramming. On the other hand, HIF1 absence in normoxia is not able to induce *PKM2* expression or glycolytic profile establishment, which makes PKM2 activation useless.

Regarding *PPARγ*, we also found no effect of the agonist ciglitazone on PGC reprogramming. *PPARγ* is a direct target of HIF1 and vice versa; therefore, *PPARγ* and *HIF1* might establish a positive loop that could cause an enhancement of glycolysis. However, in normoxic conditions in which no HIF1*α* is detected, PPAR*γ* would be incapable of creating a glycolytic profile. Our data showing that a PPAR*γ* agonist does not improve PGC reprogramming in hypoxia could indicate that the metabolic circuit had already been established through HIF1 activity and PDH inhibition. Thus, enhancement of PPAR*γ* activity would have no effect on PGC reprogramming.

Taking into account that *PDK1* is a HIF1 target, it appears likely that hypoxia-induced HIF1*α* expression provokes metabolic reprogramming in low-oxygen conditions [10, 22, 23]. This same observation has been found in DCA- and resveratrol-induced reprogramming in this study. However, it is rather surprising that DCA, a *PDK1* inhibitor, induces PGC reprogramming. In particular, this effect is unexpected because *PDH* inhibition through *PDK1* action is one of the characteristics of PGC reprogramming [10]. In fact, *OSKM* induction results in an increase of *PDK1* expression in other cell types [29]. There are many examples in which DCA causes a metabolic shift toward glycolysis, leading to stem cell differentiation, a decrease in iPSC derivation efficiency, or cancer cell apoptosis [12, 30–32]. Our results also show that a high DCA dose provokes an inhibition of PGC reprogramming. However, a low DCA dose maintains a glycolytic profile, as supported by immunofluorescence and flow cytometry analysis, and induces PGC reprogramming, demonstrated by three germ layer differentiation. A plausible explanation is that at the low concentration, DCA is not inhibiting *PDK1* but is exerting another crucial event in PGC reprogramming. In fact, different cell types do not react identically to DCA addition. For instance, glioma cancer stem cells are DCA sensitive, showing an oxidative metabolic profile that causes their differentiation and death, whereas neural stem cells do not show this behavior [32, 33]. DCA can even induce an increase in proliferation, as observed in neuroblastoma cells in a mouse xenograft model [34]. Interestingly, DCA induces a greater *Oct4/PKM2* interaction [32], indicating that DCA might directly contribute to lower Oct4 levels, which might in turn promote reprogramming.

Autophagy is required for iPSC derivation, although there are discrepancies concerning the molecular pathway involved. It has been reported that a transitory *Sox2* overexpression takes place during the first days of reprogramming, leading to canonical autophagy induction, with Atg5 and LC3-II as major regulators. It has also been reported that pluripotency induction in cells lacking *Atg5* shows a reprogramming impairment [35]. However, another study established that this impairment could be reversed, augmenting *OSKM* expression, and that the essential pathway was Atg5-independent, relied on *Rab9* and *sintaxin 7*, and included AMP-kinase (AMPK) activation, endoplasmic reticulum autophagy, and mitophagy [36, 37].

PGC reprogramming shares aspects of both hypotheses. PGCs and hiEGLs are Sox2 positive, which is necessary for autophagy. In addition, HIF1 induces *Bnip3* expression, which in turn inhibits mTORC1 binding Rheb, an mTOR activator. Thus, Bnip3 triggers autophagy [38]. Our results show an important increase in *Bnip3* expression in hypoxia, which likely inhibits *mTOR* and induces autophagy. However, resveratrol's mechanism of action occurs through Sirt1 activation, an NAD^+^-dependent deacetylase [39, 40]. Because spermidine addition did not show an effect in PGC reprogramming and the resveratrol and spermidine pathways share *Atg5*, *Atg7*, and *LC3* deacetylation [41, 42], it appears likely that the autophagy observed in PGC reprogramming does not take place through the canonical pathway.

Inhibition of autophagy using chloroquine diminished the number of reprogrammed cells, indicating the importance of autophagy in PGC reprogramming. It appears logical that the energetic stress and metabolic reprogramming that takes place during PGC culture in hypoxia drives a rearrangement of cellular structures in order to adapt to a new environment; in fact, we have observed mitophagy by electron microscopy not only in hypoxia but also with DCA and resveratrol. Mitophagy logically provokes lower ROS production and, consequently, prevents cell death [43, 44]. It has been reported that autophagy inhibition in cells overexpressing Bnip3 causes cell death, given that these cells are incapable of generating, in turn, their protective response [44]. Because Sirt1 can deacetylate *forkhead box O3* (*FOXO3*), which in turn induces *Bnip3* expression [45], it is probable that resveratrol-induced autophagy occurs through this pathway. In fact, this mechanism has already been observed in rat kidney cells [46].

*PPARγ* is also important in autophagy induction. Apart from its role in energetic metabolism triggering *PKM2* expression, *PPARγ* can induce autophagy [47]. PPAR*γ* levels are also oxygen dependent, given that it is a HIF1 direct target [48]. Among PPAR*γ* targets are *HIF1α*, *PDK*, and *Bnip3* [28, 47, 48]. Thus, the observed increase in *PPARγ* in PGCs cultured in hypoxia, added to an increase in *Bnip3* expression, an increase in PDK activity and HIF1 stabilization, could establish an axis responsible for fixing a metabolic profile in which autophagy and mitophagy are essential to the adaptation to hypoxia. These observations agree with other studies in which hypoxia-induced and PPAR*γ*-induced autophagy required HIF1*α* [47, 49].

In terms of ROS, metabolic reprogramming in response to low oxygen levels prevents ROS generation from mitochondria as a result of OXPHOS [17]. Pluripotent stem cells show reduced ROS levels, preventing oxidative stress [16]. Ascorbic acid addition to the culture medium enhances iPSC induction using *OSK* and *OSKM*, whereas resveratrol did not show any effect [19]. Our results fit with these facts, because PGCs cultured in hypoxia show lower levels of ROS compared with those in normoxia after 3 days in culture [10]. However, ROS removal is not sufficient to induce PGC reprogramming, as shown in ascorbic acid experiments, but probably contributes to PGC survival in hypoxia. However, ROS might play another role in PGCs. Specifically, our study suggests that a transient and small increase in ROS in DCA cultures might stabilize HIF1*α*, consequently inducing PGC reprogramming. Once HIF1*α* is stabilized, it would induce a metabolic reprogramming, which is shown by PDH inactivation and inactive mitochondria. This peak in ROS production has also been observed in iPSC generation from human fibroblasts, immediately before HIF1 stabilization. In this case, ROS increase induces an elevation in the expression of *nuclear factor-* (*erythroid-derived 2-*) *like 2 (NRF2)*, a master regulator of response against ROS production that promotes a metabolic shift toward glycolysis via *HIF1* [50].

With respect to the epigenetic control of reprogramming, our present results show that *CREB-binding protein* (*CBP*) coactivator, p300, is not expressed in the PGCs' nuclei in normoxic cultures whereas it becomes expressed in all the reprogramming conditions after 48 h of culture. Similarly, acetylation of histones appears evident in all reprogramming conditions + normoxia and in hypoxia alone as early as after 48 h of treatment. In fact, histone acetylation has a close implication in PGC reprogramming. Similar to our results with VPA that reprograms PGCs even in normoxia in the absence of bFGF, another histone deacetylase (HDAC) inhibitor, trichostatin A (TSA) can reprogram PGCs into pluripotent EGCs also in the absence of bFGF [9]. HDAC inhibitors have been widely used in the field of cell reprogramming, since their addition to culture increases the efficiency. Specifically, VPA has been shown to display this mentioned improvement in the arising of iPSCs, even making *Klf4* and *cMyc* dispensable from the reprogramming cocktail [51], by reducing senescence of reprogrammed cells [52]. However, *CBP*-specific knockout in PGCs did not alter their histone acetylation levels [53], suggesting that histone acetylation in PGCs is dependent on other histone acetylases different than *CBP.* In addition to their histone acetylation properties, *CBP/p300* exerted an antiapoptotic effect in PGCs in vivo [53]. In our cultures, p300 expression could aid in the survival of reprogramming cells.

It is remarkable that distinct pathways that lead to reprogramming share *HIF1α* expression as a common feature. All reprogrammed cells show HIF1*α* protein stabilization after 48 h of culture, turning into HIF1*α* negative at approximately the sixth day of culture. Because PGCs in normoxia are always negative for HIF1*α*, this molecule appears to be essential for PGC reprogramming. In fact, iPSC derivation through *OSKM* induction is impaired if HIF1*α* is absent [29]. Also, ectopic expression of *HIF1α* during the reprogramming process promotes appearance of iPSC colonies [54]. In our experimental model, HIF1 is essential for both reprogramming and survival of PGCs in hypoxia. This observation might indicate that adaptation of PGCs to hypoxia involves metabolic reprogramming and cellular rearrangement through autophagy. Our results in all reprogrammed cells show a predominance of inactive mitochondria and observation of autophagic events. Our data also show that HIF1*α* is lost at approximately the sixth day of culture, indicating that metabolic reprogramming has been fulfilled by that time and HIF1*α* is then downregulated. Similarly, transient expression of HIF1*α* has also been reported in human embryonic stem cells (ESCs) cultured in hypoxia, where it only lasts for 48 h [55].

Regarding *HIF2α*, its expression was shown to be constitutive in PGCs, given that it is detectable from the beginning of the culture, even in PGCs cultured in normoxia. This observation is not surprising, considering that PGCs are the only cell type that retains Oct4 expression in 8.5 dpc embryos and that the transcription factor responsible for that expression is *HIF2* [56]. *HIF2α* is also involved in *Sox2* and *Nanog* expression, both of which are present in PGCs [55]. HIF2*α* is absent from the sixth day of culture in all reprogrammed cells. In agreement, it has been reported that *HIF2α* overexpression favors iPSC derivation if this overexpression takes place during the early phase of reprogramming and it exerts an inhibitory effect if it occurs in later phases [54]. On the other hand, human ESCs maintain the nuclear location of HIF2*α* for a longer time and cells deficient for *HIF2α* are unable to proliferate and cannot be maintained in culture [55]. This loss of HIF2*α* might explain the lack of self-renewal in our reprogrammed cells. We propose that *HIF1α* induction alters *HIF1/HIF2* balance and, once metabolic reprogramming has been accomplished, HIF2*α* does not reach its previous levels and reprogrammed cells fail to establish self-renewal. This HIF2 impairment might be the cause behind the heterogeneous Oct4 pattern and the rise of Oct4 low populations that enter the reprogramming process. It has recently been reported that it is a reduced Oct4 expression that directs a robust pluripotent state in ESCs [57] and that a higher range of Oct4 levels in ESCs causes heterogeneity in Nanog expression. This idea is also supported by redefinition of the low *Oct4* levels required for pluripotency entry as well as the higher *Oct4* levels required for differentiation [58]. Our data show that hypoxia, low DCA, resveratrol, and VPA induce a wider range of Oct4 levels in PGCs, accounting both for pluripotency induction and differentiation-prone colony formation. This HIF balance could also be involved in the cMyc negativity in our reprogrammed cells, given that *HIF1α* and *cMyc* compete for *Sp1* cofactor binding, whereas *HIF2α* allows *cMyc/Sp1* binding [59]. As shown in the results, we hypothesize that the lack of Klf4 and cMyc observed in the reprogrammed cells (except VPA where cMyc is positive) is responsible for this inability to self-renew. In turn, this lack of expression might be also due to insufficient downregulation of *Blimp1*. Reprogramming PGCs toward EGCs with classical bFGF shows a critical downregulation of *Blimp1* as a major step, which drives *Klf4* and *cMyc*. Although we observed a downregulation of *Blimp1* by qRT-PCR in reprogrammed PGCs, this event might occur in a lower intensity as it occurs in classical EGC derivation.

Our data collectively suggest that pluripotency and immortality are two distinctly regulated processes, the first exerted by HIF1 control of the processes of glycolysis, autophagy, and an early peak in ROS, with an intermediate downregulation of Oct4; and the second exerted by *Blimp1* downregulation, *HIF1/HIF2* balance, and expression of cMyc and Klf4. Further studies on which genes are separately responsible for these two characteristics will be of significant interest, not only for EGCs and iPSCs but for their implications in oncogenic transformation.
